# Aerosol Infection Model of Tuberculosis in Wistar Rats

**DOI:** 10.1155/2010/426035

**Published:** 2010-03-18

**Authors:** Sheshagiri Gaonkar, Sowmya Bharath, Naveen Kumar, V. Balasubramanian, Radha K. Shandil

**Affiliations:** AstraZeneca India Pvt. Ltd., Bellary Road, Hebbal, Bangalore 560024, India

## Abstract

We explored suitability of a rat tuberculosis aerosol infection model for investigating the pharmacodynamics of new antimycobacterial agents. Infection of rats via the aerosol route led to a reproducible course of *M. tuberculosis* infection in the lungs. The pulmonary bacterial load increased logarithmically during the first six weeks, thereafter, the infection stabilized for the next 12 weeks. We observed macroscopically visible granulomas in the lungs with demonstrable acid-fast bacilli and associated histopathology. Rifampicin (RIF) at a dose range of 30 to 270 mg/kg exhibited a sharp dose response while isoniazid (INH) at a dose range of 10 to 90 mg/kg and ethambutol (EMB) at 100 to 1000 mg/kg showed shallow dose responses. Pyrazinamide (PZA) had no dose response between 300 and 1000 mg/kg dose range. In a separate time kill study at fixed drug doses (RIF 90 mg/kg, INH 30 mg/kg, EMB 300 mg/kg, and PZA 300 mg/kg) the bactericidal effect of all the four drugs increased with longer duration of treatment from two weeks to four weeks. The observed infection profile and therapeutic outcomes in this rat model suggest that it can be used as an additional, pharmacologically relevant efficacy model to develop novel antitubercular compounds at the interface of discovery and development.

## 1. Introduction

Tuberculosis remains a leading cause of death worldwide [1] despite unprecedented interest in the scientific community to better understand the pathobiology and development of newer interventional therapies. In this process, animal models of infection have been a corner stone in understanding complex pathology and immunology of tuberculosis. Guinea pigs were the first animal models used to demonstrate tuberculosis disease by Koch in 1882 [2]. Since then, a variety of animal models including mice, rabbits, and nonhuman primates [3–5] have been investigated to simulate tubercular disease and associated host responses. However, none of the models can mimic the complex pathobiology seen in humans.

The mouse continues to be a preferred species for modeling tuberculosis infection as well as for screening novel anti-TB drug candidates due to practical reasons [6, 7]. Guinea pigs, rabbits, and nonhuman primates are known to be better representatives of late human disease [4, 5] but pose a challenge for drug screening due to large compound requirements and prohibitive costs.

Rats have significantly contributed to modeling of variety of human pulmonary bacterial [8], fungal [9] and viral infections [10, 11] due to an increasing availability of rat immunological reagents. In recent years, several rat strains like Lewis, American cotton rats [12], and diabetic rat strains [13] have been successfully used to develop infection model of tuberculosis, particularly for investigating pathology and immune responses [14, 15]. However, there are no reports on therapy of tuberculosis in rat infection model. Here we report the application of Wistar rat model for approximating tuberculosis infection and the effect of front-line drugs in the containment of this infection.

## 2. Material and Methods

### 2.1. Bacterial Strain

*M. tuberculosis* H37Rv ATCC 27294, a strain sensitive to all the standard antimycobacterial agents, was used for all animal infection experiments. Bacterial cultures were prepared as described previously [16].

### 2.2. Drugs and Reagents

Rifampicin (RIF), Isoniazid (INH), Pyrazinamide (PZA), Ethambutol (EMB), and Carboxymethyl cellulose (CMC) were purchased from Sigma Chemical Co. USA.

### 2.3. Animals

The Institutional Animal Ethics Committee (IAEC), registered with the Government of India (Reg. no. 5/1999/CPCSEA) approved all animal experimental protocols and usage. Male Wistar rats were purchased from Raj Biotech Pune, India. Rats (7–8 weeks old) were randomly assigned into groups of three per cage and were allowed 2 weeks acclimatisation before experimental use. Feed and water were given *ad libitum*. Infected rats were maintained in individually ventilated cages (Allentown technologies, USA) in bio containment level 3 facilities.

### 2.4. Aerosol Infection

Wistar rats were infected via the respiratory route to obtain low-grade bacillary lung infection (~100 bacilli) using a modified Madison aerosol chamber [16]. Bacterial lung loads were estimated to determine suitable infection conditions for drug efficacy experiments. After infection, the animals were housed for the duration of the study in a bio-safety level 3 facilities. By using microbial enumeration as the dependent variable, the number of animals required per treatment group was as low as three in experiments for drug evaluation. The course of mycobacterial infection was monitored by enumeration of colony forming units (CFU) from excised lungs at 1, 2, 3, 4, 6, 8,13, and 18 weeks postinfection.

### 2.5. In Vivo Dose-Response Studies

In a separate experiment, starting 4 weeks postinfection, 3 animals per group were administered by oral gavage once daily with a dose range of front-line anti-TB drugs in a 0.25% (w/v) carboxymethyl cellulose suspension formulation. Rifampicin (30–270 mg/kg), Isoniazid (10–90 mg/kg), Pyrazinamide (300–1000 mg/kg), and Ethambutol (100–1000 mg/kg) were administered for two or four weeks. At the onset and 24 h after the completion of treatment, groups of rats were killed by exposure to CO_2_ and the lungs were aseptically removed. Left lung lobe was processed for CFU estimation to monitor drug efficacy while the right lobe was fixed in 10% formaldehyde solution for histopathology. The left lung lobe was homogenized in a final volume of 3 mL by using Teflon-Glass tissue grinders (Wheaton Inc.). Each suspension was serially diluted in 10-fold steps, and at least 3 dilutions were plated onto Middlebrook 7H11 agar supplemented with 10% albumin dextrose catalase (Difco Laboratories) and incubated at 37°C with 5% CO_2_ for 3 weeks.

### 2.6. Histopathology

The right lung lobe of each animal was removed for histopathological studies. They were infiltrated with and collected in 10% buffered neutral formalin, followed by standard histopathological processing techniques [15] using different gradation of alcohol. Paraffin embedded tissues were sectioned to 5 *μ*m thickness and stained with either Haematoxyline and Eosin stain or Ziehl Neilson staining for acid fact bacilli using quick staining kit (Becton-Dickinson).

### 2.7. Statistical Analysis

The colony counts obtained from plating were transformed to log_10_(*x*+1), where *x* equals the total number of viable tubercle bacilli calculated to be present in a given sample. Prism software version 4 (GraphPad Software, Inc., San Diego, California) was used for all the calculations.

## 3. Results

### 3.1. Course of *M. tuberculosis* Infection in Rat Lung

**Figure 1:**
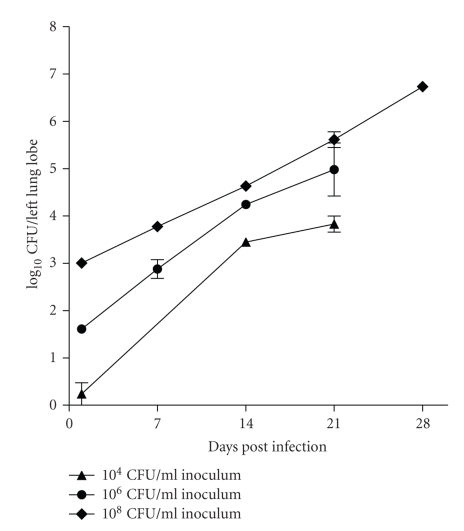
Standardisation of *M. tuberculosis* aerosol infection in Wistar rats: initial bacillary load and course of infection observed over a period of 4 weeks, following challenge with three different infection inocula.

**Figure 2:**
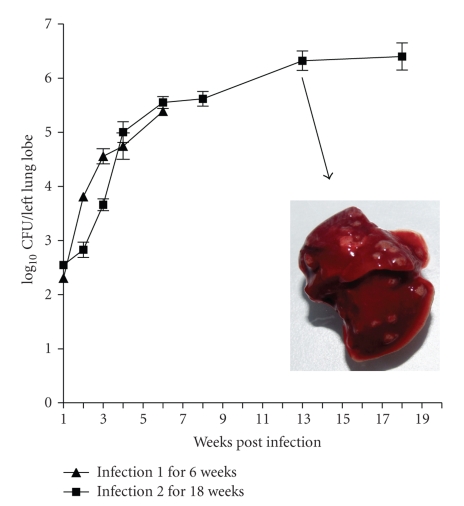
Course of infection of *M. tuberculosis* H37Rv in Rat lungs following low-grade aerosol infection. (▴): Course of infection and CFU obtained during weeks 1–7 in study 1. (■): Course of infection and CFU obtained over weeks 2–18 in study 2. Drug treatment started at week 4 postinfection. Inset shows a rat lung at thirteen-week post infection with multiple macroscopic nodular granulomas all the lung lobes following aerosol infection with *M. tuberculosis. *

The focus of initial experiments was to establish an optimum inoculum size required to consistently achieve low bacterial numbers (100–300 CFU) in the lungs. *M. tuberculosis *inocula of three different bacterial strengths (10^4^ CFU mL^−1^), 10^6^ CFU mL^−1^ and 10^8^ CFU mL^−1^) were aerosolised in a 25 mL volume using a Collison Nebulizer (BGI Incorporated, Waltham, MA) for a fixed duration, and the course of infection was monitored till four weeks. All three inocula lead to installation of bacilli into the lungs in a dose-dependent manner (Figure 1). An inoculum of 10^6^ CFU mL^−1^ consistently delivered ~200 bacilli/lung and was considered optimum for further experiments. The course of infection following an initial load of 10^8^ CFU mL^−1^ was steep, while that following 10^4^ CFU mL^−1^ yielded inconsistent infection across animals. Thus these two inocula were not preferred for further experiments. The course of tuberculosis infection increased logarithmically up to the first 6 weeks. The growth rate declined thereafter and the net bacterial load in the lungs increased by merely 1 Log_10_CFU over the next 12-week period (Figure 2).

### 3.2. Gross and Histopathology Findings

Grossly the rats developed circumscribed granuloma of varying sizes (pinpoint to 3 mm in diameter) distributed over the lung surface by 6 weeks and gradually increased to 5 mm and showed raised appearance (Figure 2 inset). Histopathological examination of the granuloma at various intervals during the course of infection revealed strong association between bacillary loads and pathology. Microscopically, for the first two weeks there were no histopathological changes in the lungs except for mild inflammatory response in the blood vessel. By week 2, there was mononuclear cell infiltration in alveolar spaces distributed sparsely in lower inoculum (10^4^ CFU mL^−1^) and extensively in higher inoculum groups (10^6^ CFU mL^−1^ and 10^8^ CFU mL^−1^). By 4 weeks, granulomatous lesions were seen in lungs across all groups (Figure 5), although the number of such foci varied depending on the initial inoculum. The predominant cell types were macrophages/histiocytes and foam cells. Lymphocytic aggregation was predominant while few epitheloid cells and multinucleated histiocytes were seen occasionally. Some degenerating neutrophils were also seen. There was no central zone of necrosis and peripheral fibrosis. The general architecture of granuloma resembled that seen in mice.

### 3.3. Bactericidal Activity of Drugs

**Figure 3:**
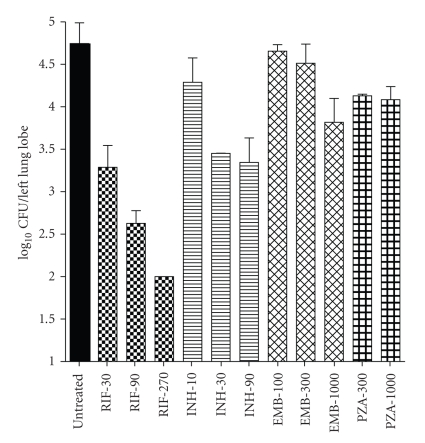
Dose response of front-line TB drugs in rats following two-week treatment. log_10_ CFU counts/left lung lobe plotted against a range of drug regimens. Rifampicin (30, 90 and 270 mg/kg), Isoniazid (10, 30, and 90 mg/kg), Ethambutol (100, 300, and 1000 mg/kg) and Pyrazinamide (300 and 1000 mg/kg) were dosed per oral once daily for two weeks (12 doses). Each bar represents the mean CFU counts from three animals.

The dose response of four frontline TB drugs was determined in the rat infection model over a wide concentration range administered once daily per-orally for 2 weeks. RIF exhibited most potent and a sharp dose-dependent bactericidal activity among all drugs tested (Figure 3). RIF 30, 90, and 270 mg/kg doses resulted in 1.5, 2.1, and 2.7-log_10_ CFU reductions, respectively, in the lungs following two weeks of treatment. INH was the second most efficacious drug with 0.4, 1.2, and 1.3-log_10_ CFU reductions, respectively, at 10, 30, and 90 mg/kg doses. EMB was less efficacious than RIF and INH but exhibited a clear dose response. The net bactericidal effect of EMB 100, 300 and 1000 mg/kg was 0.1, 0.2 and 0.9-log_10_ CFU reductions, respectively. In contrast, PZA had activity of 0.6 and 0.7-log_10_ CFU reductions at 300 and 1000 mg/kg doses.

**Figure 4:**
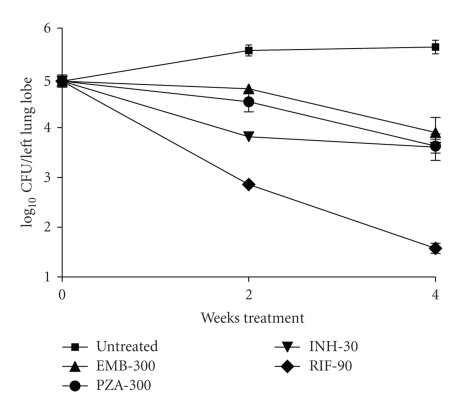
Time course of effect of front-line TB drugs on rat lung infection (RIF-90 mg/kg; INH-30 mg/kg; EMB 300 mg/kg and PZA 300 mg/kg). Drug treatment was given per oral once daily for 2- and 4-week periods.

**Figure 5:**
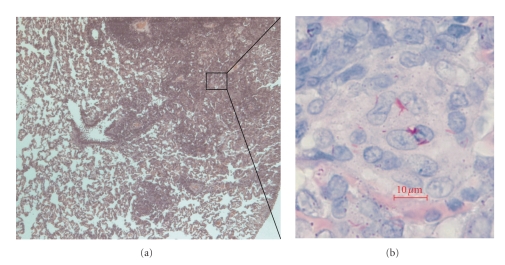
H&E at 100× magnification (a) and ZN stain at 1000× magnification (b) of a lung granulomatous lesion at 4 weeks post infection.

In a second experiment, we compared the effect of RIF 90 mg/kg, INH 30 mg/kg, EMB 300 mg/kg, and PZA 300 mg/kg administered per-orally once daily either for 2 weeks or 4 weeks. The effect was reproducible across the two experiments (Table 1); RIF, EMB, and PZA exhibited significantly higher bactericidal activity when the duration of treatment was increased from 2 to 4 weeks, while INH did not (Figure 4).

## 4. Discussion

Animal models are an integral part of drug discovery programs. They allow understanding of disease process and evaluation of new interventions in a dynamic system thus providing a link between in vitro potency and therapeutic use.

Traditionally, mice have been the model of choice for early preclinical testing of drug candidates for antitubercular activity because of their relative ease in handling, lower maintenance costs, and need for small amounts of experimental drugs to study pharmacokinetics and pharmacodynamics [6, 7, 17]. In contrast, rats have been considered unsuitable for experimental tuberculosis since they were noted to be resistant to tubercle bacilli. Neither high doses of tubercle bacilli given parenterally could kill nor produce necrotic tuberculous lesions and tuberculin sensitivity. 

The pathology observed in mice following *M. tuberculosis* infection does not entirely reflect the human disease. In contrast, larger animals like guinea pigs, rabbits, and nonhuman primates better approximate human tuberculosis [18–21]. However, the obvious limitation for drug screening remains the larger size, logistics and drug quantity requirements. In recent years, evidence is available where tuberculosis infected rats have necrotic lesions and chronic infections in the lungs [13, 14, 22], that are closer to the events observed in human disease than in the mice model. Thus, rats may provide an intermediate option in terms of representing more histopathological aspects of human disease than mice, yet not posing significantly higher demands on logistics and drug substance.

We report a Wistar rat aerosol infection model of tuberculosis suitable for investigating the pharmacodynamics of antitubercular drugs. Three key reasons that prompted us to explore a rat infection model are as follows.

1. Rats are widely accepted species for investigating pharmacokinetics and toxicokinetics at the preclinical and development stages of drug discovery programs [15].
2. In recent years, there are many reports on rat *M. tuberculosis* infection models for investigating immunology and pathology of tuberculosis [12–15, 19]. A report indeed showed that granulomas in *M. tuberculosis* infected American cotton rats [12] exhibit caseous central necrosis similar to humans thereby adding additional value to the animal model.
3. We reasoned that if the response of *M. tuberculosis* infected rats to antitubercular therapy is reproducibly established then the entire PK, PD, and toxicological investigations at the interface of discovery and development can be done in the same species.

We have reproducibly achieved chronic rat lung infections similar to that reported in American Cotton rats [12] and Lewis rats [14]. In our study, infected rats appeared healthy during the 18 weeks of observation. Aerosol infection allowed us to mimic portal of entry as in humans, resulting in inflammatory disease leading to pulmonary granulomas. The biphasic nature of infection may provide an opportunity to study drug effects on actively replicating bacilli (week 1–6) or on minimally replicating bacilli (week 7–18). 

The histopathological changes observed in Wistar rats over a period of 18 weeks had no central necrosis similar to mice [7] and other rat strains like type 1 diabetic rats [13] and Lewis rats [14]. In contrast, American cotton rats [12] and F344/N-rnu nude rats [22] have been shown to exhibit central necrosis within granulomas suggesting variable immunological and pathological outcomes across different rat strains.

Our study established efficacy of four front-line TB drugs (RIF, INH, PZA, and EMB) in the Wistar rat infection model. Significant bactericidal activities of four reference drugs acting on distinct molecular targets undoubtedly suggest suitability of rat infection model for screening of compounds with antitubercular activity. Thus, Wistar rat model offers a significant advantage over the mouse model since it presents histopathological changes that are closer to that observed in human tuberculosis, while simultaneously permitting the study of pharmacokinetics, pharmacodynamics, and safety pharmacology in the same species. This would provide a better handle in terms of predicting the human dose with the appropriate safety margins.

## Figures and Tables

**Table 1:** Reproducibility of infection and response to treatment across 2 independent experiments. Each data point represents the mean ± SD data from three animals per group.

				log_10_ CFU/Lung		
		Untreated	RIF-90	INH-30	EMB-300	PZA-300
						
Expt.1	Mean	4.75	2.63	3.45	4.51	4.13
	Std. Dev.	0.42	0.21	0.01	0.39	0.03
Expt.2	Mean	4.94	2.86	3.82	4.78	4.52
	Std. Dev.	0.21	0.11	0.03	0.02	0.35
